# Supplementary material for: Role of Molecular Weight in Polymer Wrapping and Dispersion of MWNT in a PVDF Matrix
Source: Polymers (Basel). 2019 Jan 17;11(1):162. doi: 10.3390/polym11010162 (PMC6401810; doi:10.3390/polym11010162)
Supplement: Supplementary file 1 [file polymers-11-00162-s001.pdf]

Role of Molecular weight in Polymer wrapping and dispersion of MWNT in a PVDF matrix

Muthuraman Namasivayam,[1] Mats R Andersson[1,*] and Joseph Shapter[1, 2*]

[1] Flinders Centre for Nanoscale Science and Technology, College of Science and Engineering, Flinders University, Bedford Park, Adelaide, South Australia 5042, Australia

[2] Australian Institute for Bioengineering and Nanotechnology, The University of Queensland, St. Lucia, Brisbane, Queensland 4072, Australia

*Correspondence: j.shapter@uq.edu.au or mats.andersson@flinders.edu.au

Supplementary Materials

Table S1: Thermal Properties of PVP_{10000} functionalised MWNT-PVDF composite

Conc. Of PVP_{10000} (wt. %)	ΔH_m (J g^{-1})	X_c (%)	T_m (°C)
2.44 %	39.83	42.78	168.60
9.09 %	39.24	42.15	168.05
16.67 %	35.74	38.39	165.71
33.34 %	33.63	36.12	168.04

Table S2: Thermal Properties of PVP_{40000} functionalised MWNT-PVDF composite

Conc. Of PVP_{40000} (wt. %)	ΔH_m (J g^{-1})	X_c (%)	T_m (°C)
2.44 %	32.45	34.85	166.78
9.09 %	36.96	39.70	168.25
16.67 %	39.48	42.41	167.24
33.34 %	37.15	39.90	167.72

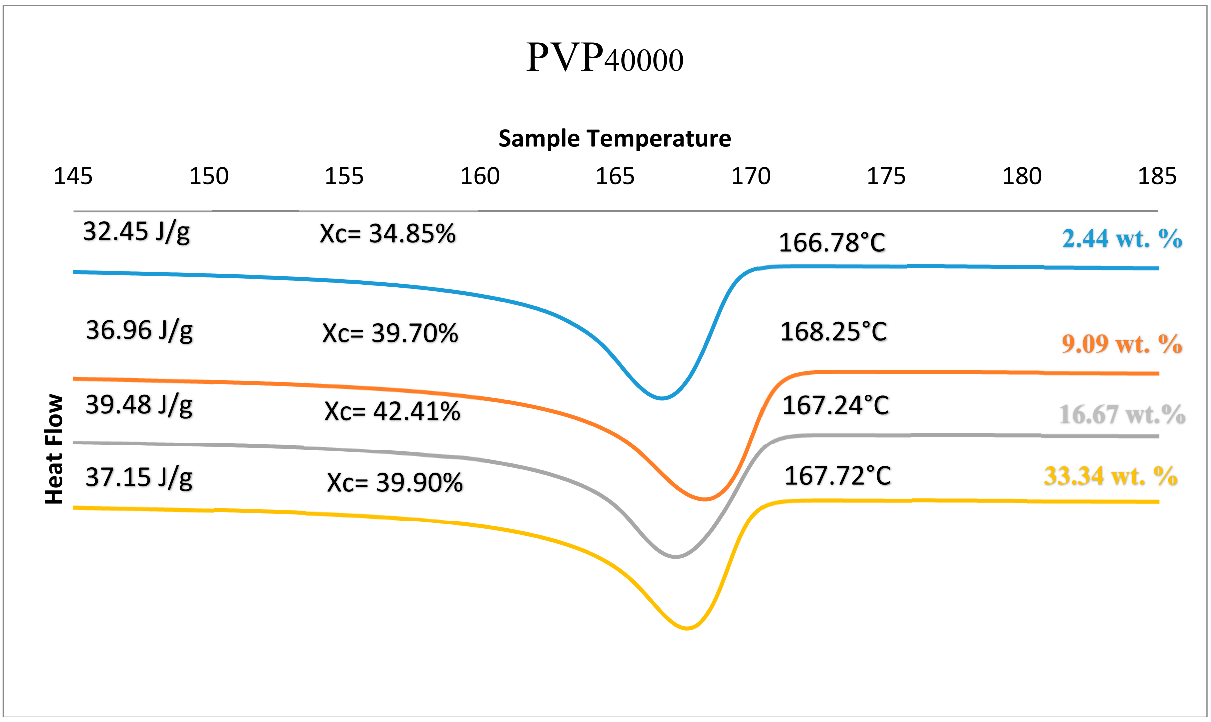

Figure S1. Thermal Properties of PVP_{40000} functionalised MWNT/PVDF composite.

Table S3: Thermal Properties of PVP_{55000} functionalised MWNT-PVDF composite

Conc. Of PVP_{55000} (wt. %)	ΔH_m (J g^{-1})	X_c (%)	T_m (°C)
2.44 %	37.11	39.86	167.89
9.09 %	36.18	38.86	167.71
16.67 %	37.79	40.59	167.72
33.34 %	36.21	38.89	167.21

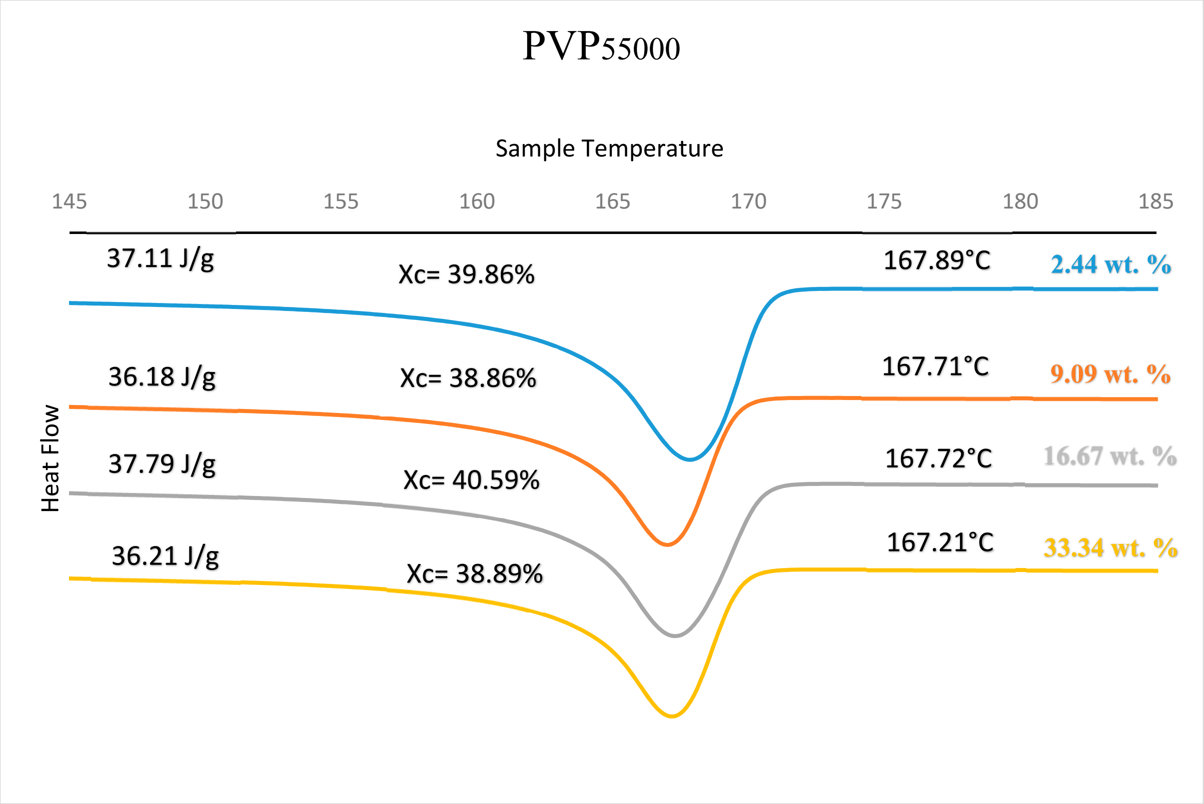

Figure S2. Thermal Properties of PVP_{55000} functionalised MWNT/PVDF composite.
